# Pre-emptive paracetamol reduces intra-operative opioid use in patients undergoing day-case oncologic breast surgery

**DOI:** 10.17179/excli2023-6804

**Published:** 2024-02-29

**Authors:** Daniah Alsaadi, Lyndon Low, James Ting, Michael Craughwell, John McDonnell, Aoife Lowery, Karl Sweeney

**Affiliations:** 1 Breast and Endocrinology Surgical Department, University Hospital Galway, Galway, Ireland

**Keywords:** pre-emptive analgesia, opioid-sparing, intra-operative opioids, pain, breast surgery

## Abstract

Minimization of intra-operative opioid use is an area of ongoing research interest with several potential benefits to the patient. Pre-emptive analgesia, defined as the administration of an analgesic before surgery to prevent establishment of central sensitization of pain, is one avenue that has been explored to achieve this. A retrospective observational study was undertaken to examine the effect of pre-emptive paracetamol on intra-operative opioid requirements. The medical and operative data of 156 patients who underwent day-case wide local excision and sentinel lymph node biopsy with and without regional block surgery at our center between October 2019 and May 2022 was carried out. Data were collected on demographics, total intra-operative and immediate post-operative opioid consumption. 57 patients did not receive pre-emptive paracetamol while 90 did. Baseline characteristics were similar. Our results showed a statistically significant reduction in morphine (p <0.029) and remifentanil (p <0.007) consumption in patients who received a regional block and pre-emptive paracetamol. Those who did not receive a regional block and were given pre-emptive paracetamol had a decrease in OxyNorm (p <0.022) requirements. A combination of general anesthesia (GA), regional block and pre-emptive paracetamol reduced intra-operative consumption of Fentanyl, OxyNorm, diclofenac, dexketoprofen, and clonidine (P <0.001) when compared to just GA alone. Use of pre-emptive paracetamol in reduction of intra-operative opioid requirements showed promising results but larger studies may strengthen the evidence for this association. A multimodal analgesic approach that utilizes pre-emptive paracetamol can be a viable method to decrease intra-operative of analgesic requirements.

## Introduction

Opioids have long played a central role in the management of acute and malignancy pain. Their efficacy in the acute setting is offset by a recognized constellation of adverse effects, including nausea, vomiting and constipation (Kim et al., 2020[12]). An increasing trend in their use has been observed globally, with an associated rise in mortalities and morbidities. Unnecessary use of opioids in patients where suitable alternatives are available and benefits are less likely to outweigh risks has been noted as one of the primary causes of the opioid crisis (Moriarty et al. 2022[15]). In addition, surgery has been described as a risk factor for chronic opioid use, with some papers reporting overuse in opioid-naïve patients (3-15 %) for prolonged periods (Sun et al., 2016[20]; Jivraj et al., 2020[9]; Moriarty et al., 2022[15]). 

The ongoing opioid epidemic in the United States initiated efforts to identify opioid-sparing analgesic regimes in various settings, with over 300 publications on the topic catalogued on PubMed over the past 10 years (Shanthanna et al., 2021[19]). Reducing post-operative opioid use and prescribing has been heavily researched and advocated for (Nuckols et al., 2014[17]; Wetzel et al., 2018[23]; Daoust et al., 2022[4]). However, reducing opioid usage in the peri-operative period remains a novel area of continuing research, with pre-emptive analgesia promoted as a possible way to achieve this aim. Pre-emptive paracetamol administration has been associated with a decrease in opioid consumption 24 hours post-operatively and minimized intra-operative anesthetic requirements, yet its use remains controversial (Ali and Siddiqui, 2012[3]; Fenlon et al., 2013[6]; Khalili et al., 2013[11]; Abdelmageed and Al Taher, 2014[1]; Medina Vera and Novoa, 2016[14]; Ng et al., 2019[16]; Kim et al., 2020[12]). This study examined the impact of pre-emptive paracetamol use on intra-operative opioid requirements in patients undergoing oncologic breast surgery. 

## Methods

A retrospective observational case series study was conducted at our hospital from October 2019 to May 2022. Adult females undergoing elective day-case unilateral wide local excision and sentinel lymph node biopsy were included. Patients undergoing bilateral wide local excision with sentinel lymph node biopsy, wide local excision with axillary clearance or a mastectomy with or without an axillary procedure were excluded, as they were patients who had to stay overnight following their procedure. 

Data were collected on the patients' demographics, whether or not pre-emptive analgesia in the form of 1 g of paracetamol via the oral route was administered, whether or not a successful regional anesthetic block was administered, and whether or not the patient required opioids in the intra-operative and immediate (within four hours) post-operative period. Additionally, the agent, dose and route of administration of opioids were documented if it was required, and we collected data about the surgical and anesthetic teams caring for each patient. Statistical analysis was performed using GraphPad Prism 9, version 9.2.0 (GraphPad Software, Inc., San Diego, CA). A one-tailed Fischer's exact test with a confidence interval of 95 % was used in place of the chi-squared test as >20 % of cells in the contingency table had < 5 expected counts. P-values less than < 0.05 were considered significant. This study adhered to the Preferred Reporting of Case Series in Surgery (PROCESS) guidelines (Agha et al., 2018[2]).

## Results

A total of 147 consecutive patients were included: the control group (n=57) and the pre-emptive paracetamol group (n=90, intervention group). The mean age was 80 and 79 years, respectively. There was an equal distribution of right and left-sided surgery performed in both groups, and all patients refrained from eating or drinking for at least eight hours before the surgery. All patients in the intervention group received 1 g of pre-emptive paracetamol orally within four hours prior to surgery and none of the patients in the control group did. A regional paravertebral block consisting of 0.75 % ropivacaine 20 ml, 1 % lignocaine 10 ml and dexamethasone 8 g was administered under ultrasound guidance based on the primary anesthesiologist's preference and patient's characteristics. 

**Figure 1:**
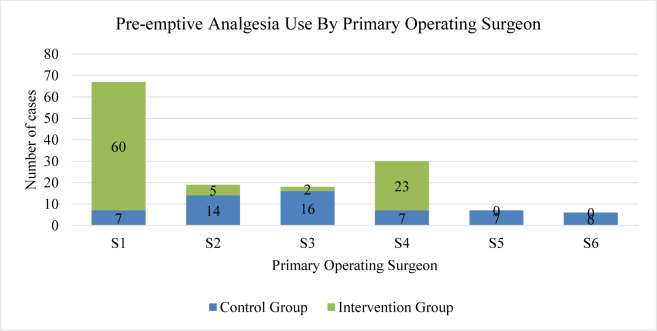
Pre-emptive analgesia use by primary operating surgeon

**Figure 2:**
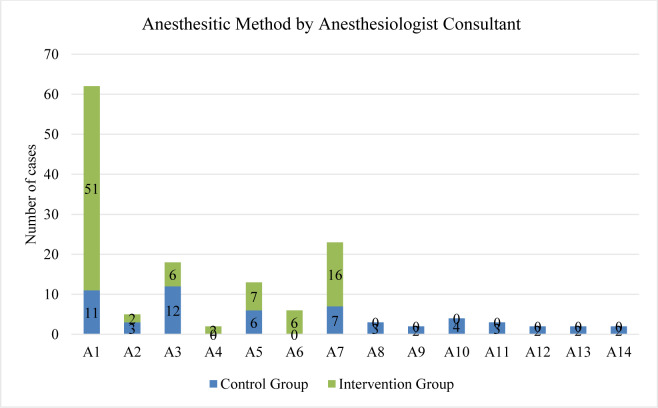
Anesthesiologist consultant cases and their use of regional blocks

The peri-operative team included six primary operating surgeons and 14 anesthesiologists. Variable use of pre-emptive analgesia by surgeons was noted (Figure 1(Fig. 1)); it was shown that surgeons 1 and 4 had the highest rates, 90.7 % and 76.6 %, respectively. Figure 2(Fig. 2) displays the anesthetists' total number of cases with their use of regional blocks; anesthesiologists 1 and 7 used regional blocks in 90 % and 88.8 % of their cases, respectively. A regional paravertebral block, which required an additional 30 minutes on average, was administered in 15/57 (26 %) patients in the control group and 79/99 (80 %) patients in the intervention group. In addition, whenever given, a local anesthetic was administered at the time of closure.

**Figure 3:**
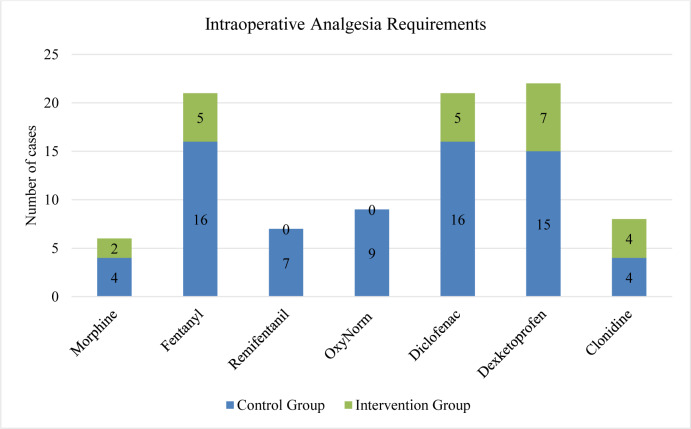
Overall intra-operative analgesia requirements

Overall, the intra-operative requirements of non-steroidal anti-inflammatory drugs (NSAIDs) and opioids in the intervention group were decreased when compared to the control group (Figure 3(Fig. 3)). Patients that received pre-emptive paracetamol with the regional block had decreased Morphine and Remifentanil requirements with a statistical significance of p <0.029 and p <0.007, respectively when compared to regional block-only patients (Table 1(Tab. 1)). The remainder of NSAIDs and opioid demands were reduced but were not statistically significant. 

The cohort of patients that received a general anesthetic (GA) without a regional block were compared in Table 2(Tab. 2); those that received pre-emptive paracetamol had a reduction in OxyNorm requirements with a p-value of <0.022. The remainder of NSAIDs and opioid requirements decreased but were not statistically significant. The selected group of patients that received both pre-emptive paracetamol and a regional block was compared to the GA-only group (Table 3(Tab. 3)); The administration of fentanyl (P <0.001), OxyNorm (P <0.001), diclofenac (P <0.001), dexketoprofen (P <0.003), and clonidine (P <0.018), was reduced with a statistical significance as shown. Overall, immediate post-operative opioid requirements decreased by 1 % in the intervention group (39.4 % vs 40.4 %, P = 0.89). Length of stay was not impacted by opioid use in all our patient cohorts as all day cases were discharged on the same day without any immediate side effects or requirement for anti-emetic or other medications, although we were unable to account for the exact discharge time. 

See also the Supplementary data.

## Discussion

The introduction of multimodal analgesia regimens resulted in enhanced pain control, reduced opioid consumption peri-operatively, expedited post-operative recovery and decreased opioid prescriptions upon discharge. The introduction of pre-emptive analgesia has further strengthened these findings (Elvir-Lazo and White, 2010[5]; Trabulsi et al., 2010[21]; Kim et al., 2020[12]; George et al., 2023[7]). A retrospective study of 60 patients that underwent a robotic-assisted laparoscopic radical prostatectomy investigated the impact of multimodal analgesia (pregabalin 150 mg, acetaminophen 975 mg, and celecoxib 400 mg) on the intra-operative consumption of opioids. The intervention group (n = 30) had a significant reduction in intra-operative morphine requirements (38.4 ± 2.73 mg vs 49.1 ± 2.65 mg; P < .01) (Trabulsi et al., 2010[21]). 

Paracetamol, a metabolite of phenacetin, is known for its non-opioid analgesic and antipyretic effects. Its mechanism of action remains unclear, but it is accepted that it works centrally and peripherally to inhibit the synthesis of prostaglandin by cyclooxygenases 1 and 2. Additionally, more recent studies revealed that paracetamol functions through the descending serotonergic pathway and spinal 5-HT receptors to prevent central nociception (Karaman, 2016[10]; Medina Vera and Novoa, 2016[14]; Ng et al., 2019[16]). Pre-emptive paracetamol has been extensively researched and shown to reduce post-operative pain scores and opioid consumption (Medina Vera and Novoa, 2016[14]; Ng et al., 2019[16]; Xuan et al., 2021[24]). A prospect study published in 2020 proposed pain management guidelines for oncologic breast surgery based on a systematic review of 749 studies and a meta-analysis of 53 studies. It was recommended to use peri-operative paracetamol for continuous systematic analgesia instead of opioids (Jacobs et al., 2020[8]). Interestingly, a randomized, double-blind, placebo-controlled study of 62 patients revealed that pre-emptive paracetamol decreased sevoflurane requirements intra-operatively (36.2 ± 15 vs 44.9 ± 13.9 ml in the control group; p = 0.021) and resulted in a faster post-anesthetic recovery profile (Abdelmageed and Al Taher, 2014[1]). Studies have reported variable results as to whether intravenous and oral route paracetamol peak plasma concentrations are similar. However, the majority of the studies stated that peak plasma concentrations are greater and achieved quicker after intravenous administration rather than oral (Van Der Westhuizen et al., 2011[22]; Langford et al., 2016[13]). On the other hand, some papers argued that the difference between administration routes is insignificant and that the oral route can reduce the additional costs and risks attached to intravenous preparation (Fenlon et al., 2013[6]; Patel et al., 2020[18]). 

To our knowledge, our study was one of the first to examine the intra-operative impact of a single dose of pre-emptive paracetamol for elective oncologic day-case breast procedures. The results revealed a statistically significant reduction in morphine and remifentanil consumption in patients who received a regional block and pre-emptive paracetamol. Patients who did not receive a regional block and were given pre-emptive paracetamol had a statistically significant decrease in OxyNorm requirements. A combination of GA, regional block and pre-emptive paracetamol demonstrated reduced intra-operative analgesic demands for fentanyl, OxyNorm, diclofenac, dexketoprofen and clonidine compared to GA alone. Certainly, with these results in mind, future research may look at using a combination of pre-emptive non-opioid analgesia and paravertebral block, without any use of intraoperative opiates.

This study was limited by sample size and confided to breast oncologic day-case surgeries. It was retrospective and a single primary surgeon and anesthetist performed most cases; thus, bias and influence cannot be excluded. Furthermore, due to the retrospective nature of the paper, titration of intraoperative opiates to the depth of anesthesia or to any intraoperative hemodynamic parameters were not looked at. 

## Conclusion

In summary, the use of pre-emptive paracetamol to reduce intra-operative opioid requirements showed promising. However, more extensive studies and randomized clinical trials are needed to further explore and examine this relationship. In addition, this review identified that a multimodal analgesic approach that utilizes pre-emptive paracetamol is a viable method to decrease the intra-operative analgesics requirements.

## Declaration

### Acknowledgments 

Not applicable.

### Conflict of interest 

The authors have no conflicts of interest to disclose. 

### Funding 

The authors have no relevant financial or non-financial interests to disclose.

### Author contributions 

Conceptualization, methodology - Daniah Alsaadi.

Formal analysis and investigation - Daniah Alsaadi, Lyndon Low, James Ting, Michael Craughwell.

Writing - original draft preparation - Daniah Alsaadi.

Writing - review and editing - Daniah Alsaadi, Lyndon Low.

Supervision - John McDonnell, Karl Sweeney, Aoife Lowery.

## Supplementary Material

Supplementary data

## Figures and Tables

**Table 1:**
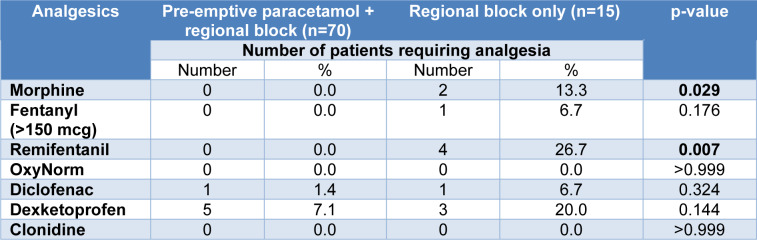
Pre-emptive paracetamol with regional block versus regional block only

**Table 2:**
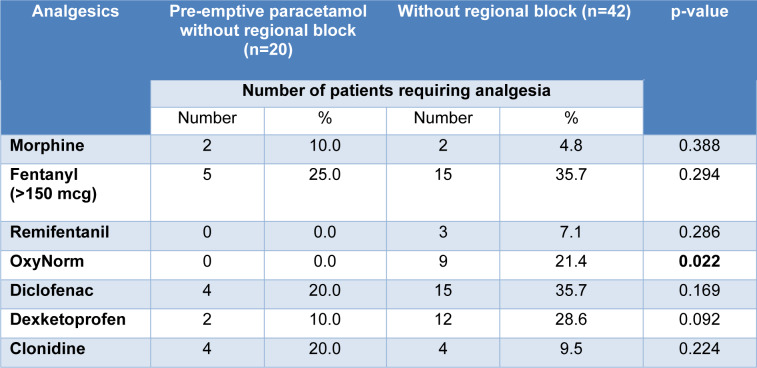
Pre-emptive paracetamol without regional block versus control group

**Table 3:**
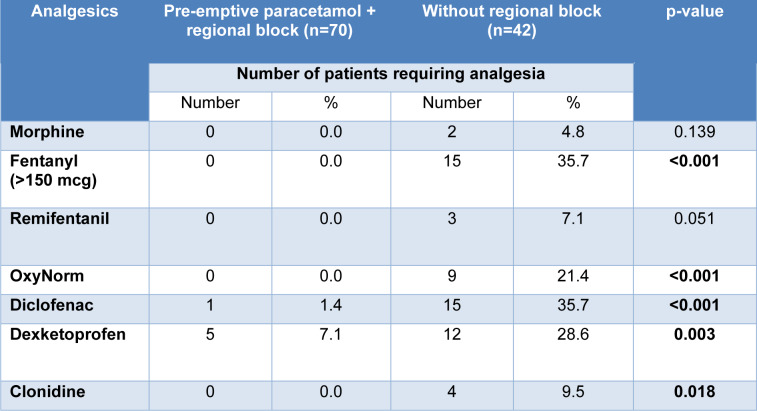
Pre-emptive paracetamol with regional block versus control group

## References

[1] Abdelmageed WM, Al Taher WM (2014). Preoperative paracetamol infusion reduces sevoflurane consumption during thyroidectomy under general anesthesia with spectral entropy monitoring. Egypt J Anaesth.

[2] Agha RA, Borrelli MR, Farwana R, Koshy K, Fowler AJ, Orgill DP (2018). The PROCESS 2018 statement: Updating Consensus Preferred Reporting Of CasE Series in Surgery (PROCESS) guidelines. Int J Surg.

[3] Ali M, Siddiqui S (2012). Is intravenous paracetamol a useful adjunct for intraoperative pain ?. Health (Irvine Calif).

[4] Daoust R, Paquet J, Marquis M, Chauny JM, Williamson D, Huard V (2022). Evaluation of interventions to reduce opioid prescribing for patients discharged from the emergency department: a systematic review and meta-analysis. JAMA Netw Open.

[5] Elvir-Lazo OL, White PF (2010). The role of multimodal analgesia in pain management after ambulatory surgery. Curr Opin Anaesthesiol.

[6] Fenlon S, Collyer J, Giles J, Bidd H, Lees M, Nicholson J (2013). Oral vs intravenous paracetamol for lower third molar extractions under general anaesthesia: Is oral administration inferior?. Br J Anaesth.

[7] George M, N K, M R (2023). Effect of Preemptive multimodal analgesia regimen on post-operative epidural demand boluses in lower limb orthopaedic surgeries. Cureus.

[8] Jacobs A, Lemoine A, Joshi GP, Van de Velde M, Bonnet F, Pogatzki-Zahn E (2020). PROSPECT guideline for oncological breast surgery: a systematic review and procedure-specific postoperative pain management recommendations. Anaesthesia.

[9] Jivraj NK, Raghavji F, Bethell J, Wijeysundera DN, Ladha KS, Bateman BT (2020). Persistent postoperative opioid use - a systematic literature search of definitions and population-based cohort study. Anesthesiology.

[10] Karaman T (2016). Comparison of the analgesic effects of preemptive lornoxicam and paracetamol after laparoscopic cholecystectomy. Int J Anesth Anesthesiol.

[11] Khalili G, Janghorbani M, Saryazdi H, Emaminejad A (2013). Effect of preemptive and preventive acetaminophen on postoperative pain score: a randomized, double-blind trial of patients undergoing lower extremity surgery. J Clin Anesth.

[12] Kim MP, Godoy C, Nguyen DT, Meisenbach LM, Chihara R, Chan EY (2020). Preemptive pain-management program is associated with reduction of opioid prescriptions after benign minimally invasive foregut surgery. J Thorac Cardiovasc Surg].

[13] Langford RA, Hogg M, Bjorksten AR, Williams DL, Leslie K, Jamsen K (2016). Comparative plasma and cerebrospinal fluid pharmacokinetics of paracetamol after intravenous and oral administration. Anesth Analg.

[14] Medina Vera A, Novoa LM (2016). Decrease anesthetic requirements and analgesics postop, in patients undergoing laparoscopic cholecystectomy: premedication with acetaminophen versus intravenous ketorolac. J Anesth Crit Care Open Access.

[15] Moriarty F, Bennett K, Fahey T (2022). Opioid and analgesic utilization in Ireland in 2000 and 2015: A repeated cross-sectional study. Pharmacol Res Perspect.

[16] Ng QX, Loke W, Yeo WS, Chng KYY, Tan CH (2019). A meta-analysis of the utility of preoperative intravenous paracetamol for post-caesarean analgesia. Medicina (Kaunas).

[17] Nuckols TK, Anderson L, Popescu I, Diamant AL, Doyle B, Di Capua P (2014). Opioid prescribing: a systematic review and critical appraisal of guidelines for chronic pain. Ann Intern Med.

[18] Patel A, Pai BHP, Diskina D, Reardon B, Lai YH (2020). Comparison of clinical outcomes of acetaminophen IV vs PO in the peri-operative setting for laparoscopic inguinal hernia repair surgeries: A triple-blinded, randomized controlled trial. J Clin Anesth.

[19] Shanthanna H, Ladha KS, Kehlet H, Joshi GP (2021). Perioperative opioid administrationa critical review of opioid-free versus opioid-sparing approaches. Anesthesiology.

[20] Sun EC, Darnall BD, Baker LC, MacKey S (2016). Incidence of and risk factors for chronic opioid use among opioid-naive patients in the postoperative period. JAMA Intern Med.

[21] Trabulsi EJ, Patel J, Viscusi ER, Gomella LG, Lallas CD (2010). Preemptive multimodal pain regimen reduces opioid analgesia for patients undergoing robotic-assisted laparoscopic radical prostatectomy. Urology.

[22] Van Der Westhuizen J, Kuo PY, Reed PW, Holder K (2011). Randomised controlled trial comparing oral and intravenous paracetamol (acetaminophen) plasma levels when given as preoperative analgesia. Anaesth Intensive Care.

[23] Wetzel M, Hockenberry J, Raval MV (2018). Interventions for postsurgical opioid prescribing: a systematic review. JAMA Surg.

[24] Xuan C, Yan W, Wang D, Mueller A, Ma H, Wang J (2021). Effect of preemptive acetaminophen on opioid consumption: A meta-analysis. Pain Physician.

